# Construction of quality of life change patterns: example in oncology in a phase III therapeutic trial (FFCD 0307)

**DOI:** 10.1186/s12955-015-0342-1

**Published:** 2015-09-22

**Authors:** Gillles Nuemi, Hervé Devilliers, Karine Le Malicot, Rosine Guimbaud, Côme Lepage, Catherine Quantin

**Affiliations:** Centre Hospitalier Universitaire de Dijon, Service de biostatistique et d’Informatique Médicale (DIM), BP 77908, 21079 Dijon, Cedex France; FFCD, Inserm, U866, Université de Bourgogne, Dijon, France; Inserm, UMR 1037/CNRS-ERL 5294, Université Toulouse 3, Toulouse, France; INSERM, CIC 1432, Dijon, France; Dijon University Hospital, Clinical Investigation Center, clinical epidemiology/ clinical trials unit, Dijon, France; Inserm UMR 1181, Biostatistics, Biomathematics, Pharmacoepidemiology and Infectious Diseases (B2PHI), University Bourgogne Franche-Comté, F-21000 Dijon, France

**Keywords:** Quality of life, Change patterns, Multiple imputation, Clustering

## Abstract

**Objective:**

Quality of life data in cancerology are often difficult to summarize due to missing data and difficulty to analyze the pattern of evolution in different groups of patients. The aim of this work was to apply a new methodology to construct Quality of Life (QoL) change patterns within patients included in a clinical trial comparing to regimen of treatment in locally advanced eosogastric cancer.

**Materials and methods:**

In this trial, QoL was assessed every 2 months by self-reported EORTC QLQ-C30 questionnaire. Physical dimension scores were analyzed. After multiple imputation of missing data, 27 statistical measures aiming to describe the variation of QoL measures among follow-up were computed for each patient. Based on these measures, patient were grouped into homogenous groups in terms of QoL variation pattern using a K-Means classification method. The mean QoL score at each time was graphically represented in each obtained pattern. Finally, clinical characteristic of patients in each pattern of QoL were described and compared.

**Results:**

The trial included 416 patients and 1023 questionnaire were collected. 74 % of patients were male with a mean ± SD age of 62 ± 11 years. 43 % of scores were missing. Patients were grouped into four classes of homogeneous QoL variation patterns. 1) a Pattern of 24 (6 %) patients showing improvement in QoL with a mean variation of +10.7 points on the 0–100 scale, 2) a Pattern of 171 (41 %) patients showing a stability 3) two Patterns of 78 (19 %) and 143 (34 %) patients respectively showing a deterioration of QoL with a mean variation of −67.2 and −67.6, respectively. There were no difference between patterns in terms of gender or age. Patients within “degradation” pattern had significantly lower performance status (*p* = 0.015), higher severe after-effects rate (*p* < 10-3) and death rate (*p* < 10-3).

**Conclusion:**

This work opens up perspectives for longitudinal data analysis with a high probability of missing values while providing a relevant graphical summary. Patterns of QoL evolution with clinical relevance may help to interpret longitudinal QoL data in Cancer studies.

**Electronic supplementary material:**

The online version of this article (doi:10.1186/s12955-015-0342-1) contains supplementary material, which is available to authorized users.

## Introduction

Gastrointestinal cancers are among the most frequent cancers in France [1, 2]. Metastatic or locally-advanced cancers have a bleak prognosis. The treatments proposed only marginally improve survival, but allow progress in terms of quality of life (QoL) [3–5]. QoL is recognized as an essential criterion for the evaluation of new treatments [6–8] and a description of the evolution of patients’ feelings in the context of aggressive therapy is becoming a systematic secondary objective in phase III trials, and in a large number prognostic studies in routine practice [9, 10].

However, the analysis of QoL in patients is a very complex process and the heterogeneity of patients’ perception of their state of health often makes it difficult for clinicians to interpret the objective results of studies. We can first of all underline the subjective nature of the information collected at different time points for each patient. Then, collecting these data currently requires the use of multidimensional measurement scales, each dimension being summarized when possible by a computed score. Finally, the repeated nature of the scores recorded per patient makes it almost impossible to avoid missing data (MD) [11, 12]. The reasons for MD are wide and varied, Rubin et al. proposed, in a theoretical framework on missing data problems [13], a classification system that is widely used in the methodological literature [14–16]. According to this system, whatever the reason for missing data, it can fit into one of the three classes of missing data mechanisms. The nature of the association between the probability of missing data and other variables defines the three so-called missing data mechanisms: 1/ A missing completely at random (MCAR) mechanism occurs when the propensity for missing data on a particular variable is unrelated to other measured variables and to the would-be values of that variable, 2/ A missing at random (MAR) mechanism which holds when the probability of missing data on a variable is related to other variables, but not to the would-be values of the incomplete variable, 3/ A missing not at random (MNAR) mechanism that occurs when the probability of missing data on a variable is related to the would-be value of that variable (i.e., outcome-dependent missingness). The impact of MD must be taken into account to attenuate the non-negligible risk of bias [15, 17].

Nonetheless, taking this subjective element (QoL) into account is particularly important with regard to the results of phase III therapeutic trials involving patients with advanced gastrointestinal cancer for whom the objective to improve QoL is essential. Therefore, modeling individual-level heterogeneity in QoL evolutions (aka trajectories) remains a goal for exploratory and prediction purpose, as does capturing the heterogeneity of individual QoL trajectories. For this, it may be of interest to identify clusters or groups of these trajectories that may reflect distinctive individual differences. These groups must be described, presented and interpreted in a way that is simple and understandable. The methods used to analyze changes in longitudinal data have been evolving steadily over the last twenty years or so and notably since the use descriptive methods, which use measurements of central trends (means, medians,…) and thus eclipse the longitudinal nature of the data. We can note, the contribution of growth curve models (also known as mixed effects models, random coefficient models, and multilevel models), which have allowed a more precise description of the data. The approach in our case would be to model population distribution of trajectories based on continuous distributions (of scale scores) in order to sort out individual factors accounting for variation concerning, for example, the population. This approach requires strong technical assumptions about distribution (multivariate normal distribution) and the results remain difficult to interpret for the non-specialist [18, 19]. Another contribution is due to the group-based trajectory models (GBTM) which extend the previous work with a qualitative approach to modeling individual difference by identifying clusters of individuals with similar trajectories. Here, individual differences that may explain or at least predict individual-level heterogeneity can be expressed in terms of groups’ differences [20]. When datasets contain MNAR data, the model specification based on the GBTM approach at its current stage of development may not accommodate such a complex missing data mechanism [20]. Today, even more sophisticated methods make it possible to identify subgroups of QoL change patterns from scale scores (quantitative variables) and to elaborate specific hypotheses in each subgroup of patients [21–23], thus making it possible to model individual-level characteristics in these patterns. These methods are becoming easier to use and provide easily interpretable results. At the same time, methods to take MD into account in the analysis of clinical studies are being standardized [18, 24–28].

The aim of this work was to use these new methods first to construct a typology of QoL change patterns (CP) in the context of a phase III therapeutic trial in patients with locally-advanced and metastatic gastrointestinal cancer, and secondly to describe identified patterns using the variables collected.

## Materials and methods

### *Study design of the* phase III clinical trial (FFCD-0307)

We worked on data from a phase III clinical trial (FFCD-0307) conducted between June 2005 and May 2010. The results of this study are published in Guimbaud et al. [29]. It was a multicenter, randomized, open, prospective trial that compared the efficacy of two sequential polychemotherapy strategies (FOLFIRI followed by Epirubicin-Cisplatin-Capecitabine (ECX) versus ECX followed by FOLFIRI) in patients with histologically confirmed, unresectable, locally advanced or metastatic gastric or esophagogastric junction adenocarcinoma.

Other inclusion criteria were age ≥18-years; measurable and/or assessable lesions according to RECIST criteria [30]; WHO performance status (PS) ≤2; ability to take oral medications; no previous palliative chemotherapy (≥6 months from adjuvant chemotherapy was allowed); ≥3 weeks from previous radiotherapy; sufficient bone marrow function; creatininemia ≤110 μmol/L; bilirubinemia ≤35 μmol/L. Exclusion criteria were a history of fluorouracil or anthracycline cardiotoxicity, cardiac or coronary deficiency; known cerebral or meningeal metastasis; other life-threatening cancer; being pregnant or breast-feeding; inability to complete the European Organisation for Research and Treatment of Cancer Quality of Life Questionnaire core 3.0 (EORTC QLQ-C30); or unable to plan regular follow-up for any reason. This trial showed that time to treatment failure (TTF) was significantly longer with FOLFIRI first-line compared to ECX first-line therapy.

### *Collection of quality of life data in the* phase III clinical trial (FFCD-0307)

The secondary objective of the phase III clinical trial (FFCD-0307) was to evaluate quality of life measured by the EORTC-QLQ-C30 self-administered questionnaire. The study design planned an evaluation of this questionnaire every 8 weeks.

### EORTC QLQ-C30 Questionnaire

The QLQ-C30 is a questionnaire with 30 items, that covers health issues, and applicable for all cancers. Of those 30 items, 17 are grouped into 5 functional scales or dimensions (physical, cognitive, role, emotional, and social functioning) and one global health status/quality of life scale. The remaining 13 items are scales related symptoms (fatigue, nausea/vomiting, pain, dyspnea, diarrhea, insomnia, appetite loss, and constipation). The questionnaire is validated in patients with gastric cancers [31, 32]. For each scale, a score was calculated in two steps according to a standardized method [32]: first, a raw score was estimated as the average of items that contributed to the scale. And then, a linear transformation was used to standardize the raw score so that scores ranged from 0 to 100. We focused our work on the physical functioning scale with scores ranging from 0 (severe debilitation) to 100 (asymptomatic/best quality of life). This scale is based on 5 items (corresponding to questions 1 to 5 of the whole qlq-c30 questionnaire) and the patients assessed for the previous week their abilities to carry out certain everyday activities such as getting dressed, taking care of personal hygiene, carrying a bag of shopping or even going for a walk (see Additional file 1: Annex 1 for the listing of the 1 to 30 items with answer options).

### Calculation of QoL change patterns

In this study, we included all of the patients previously included in the phase III clinical trial (cf. study design). However, as all of the patients did not complete the whole questionnaire, we decided to use a data imputation method to complete the missing data.

We considered a follow-up of approximately 13 months after the randomization (corresponding to the first seven measurements of the physical functioning scale score) (QLQ-C30 PF2. Hereinafter, they will be noted T_1_, T_2_, … T_7_ . An individual score patterns (ISP) was defined as the series of scores calculated at each time point for a given patient. Starting with the initial table of ISP, four steps were necessary to identify quality of life change patterns. These steps are resumed in Additional file 1: Annex 2 and described below:

- **1/**The first step was a process of data imputation [13, 25] necessary because of MD. In randomized clinical trials with QoL assessments, it may be unrealistic to regard missing data (QoL scores) as due to a not-at-random mechanism (MCAR or MAR) [11, 12]. Thus, on the assumption of an MNAR mechanism, we applied the multiple imputation (MI) method on scores [17]. We also applied an extension to the longitudinal study of the explicit multivariate regression method as our imputation procedure [15, 17, 33]. For this, the auxiliary data (related to patients) used in the model were as follows: age, time to death, treatment arm, declaration of severe side effect, time to treatment failure and previous scores. In practical terms, the multiple imputation method involves imputing each missing score several times, say *r* times. Without going into technical detail, which can be found in references [18, 24–28], the imputations are randomly drawn from a distribution conveniently derived from the data, taking into account the relationship between auxiliary variables and the relationship of each auxiliary variable with the missing patterns in the remaining ones. Since imputations are random and not deterministic, a missing score may be replaced with a different value in each of the *r* completed data sets, and therefore the *r* data sets are not equal. This operation results in *r* completed data sets, that is, *r* data sets with the same number of variables and participants as the original one but with all missing values filled in by imputation. This method may be used for the analysis of data with large amounts of missing values [18, 24–28]. One hundred datasets with complete ISP were generated (*r* = 100). This method finally encompassed the major part of patients’ quality of life measurement variability. For the main analysis, scores after death were set to zero [34, 35].
- **2/**In the second step, variability parameters were calculated for the 100 imputed datasets. Leffondre et al. in 2004 proposed 27 statistical measures of change (renamed parameters in the following) for the identification of longitudinal patterns [21]. These parameters concern: i) parameters that described the linearity of the ISP (e.g. the standard deviation, the slope of the regression line or the part of the variance explained by a linear model), ii) those reflecting non-linearity of the ISP, such as abrupt changes over short periods (e.g. the mean of successive differences between 2 consecutive scores), iii) parameters that measured the contrast between 2 defined periods in an ISP (ratio between the change before and the change after) see Additional file 1: Annex 3 for the formula of each parameter.. At this stage, we had 100 new datasets that included the variability parameters for each ISP.
- **3/**The third step build subgroups (clusters) of ISP, applying a classification method to each dataset created. An unsupervised classification technique based on the « k-means» method, using Euclidian distances was applied [27, 36]. It addresses the following objective: given a dataset of *n* units described by *m* attributes and a positive integer *k* (the number of clusters), group the *n* units into *k* clusters so that the total sum of the distances of each unit to its nearest cluster center is minimized. To be able to simultaneously manage both the number of clusters (*k*) and the number of features (*m*) to obtain the optimal partition, we used a so-called CritCF citerion. This is a function that expresses the dependency between *k* and *m* (see the complete formula in Additional file 1: Annex 4). Its goal can be summarized as follows: in the search space defined by all possible subsets of features in conjunction with a variable number of clusters, it assigns a ranking score to each partition that may be defined. CritCF takes values in range [0,1] and should be maximized in order to simultaneously obtain the best feature subset and partition [37].
- **4/**The last step was to classify each patient in the group that best reflected his/her own QoL change pattern. This objective was reached by the mean of the aggregation of the 100 classification results. The aggregation process was carried out in accordance with a published methodology [27]. A given patient was assigned to the change pattern (CP) in which he/she was most frequently classified within the 100 classifications. For each CP, a mean ISP was calculated and presented graphically.

### Analysis of sensitivity

The objective here was to evaluate the impact of the multiple imputation procedure on the final classification of a patient in one of the CP. For this we represented each CP graphically as a box plot showing the probability of each patient being assigned to one of the classes from the clustering process on each of the 100 datasets. This could allow one to observe, for example, if the patients assigned to the first CP were mainly grouped in the first cluster. In the second step, we estimated a coefficient of concordance (Adjusted Rand index) between assignments to different clusters and final assignment to the corresponding CP. This coefficient was presented with a mean and its 95 % Confidence Interval [38–40].

### Graphical description of QoL change patterns

Each QoL change pattern was described using patients’ characteristics: Demographic data (age and gender), randomization data (tumor type, location, randomization arm and World Health Organization (WHO) Performance Status), and follow-up data (TTF, declaration of serious adverse events (Yes/no), second line treatment administration (Switch Yes/no) and the status of the patient (Alive or dead). These characteristics were presented with bar charts where each bar corresponded to a modality and the height was its relative frequency [41].

Frequencies were compared using the Chi-2 test or Fisher’s exact test when appropriate. The CP were compared using the difference between the maximum and minimum score (MaxDiff which can be negative if the first value is the minimum). Continuous quantitative variables were described as means and standard deviations. For all of the statistical tests, the threshold of statistical significance was set at 0.05.

SAS version 9.3. was used for all of the statistical analyses.

## Results

The clinical trial randomized 416 patients (209 in ECX/FOLFIRI and 207 in FOLFIRI/ECX arm, respectively). Men accounted for the majority of patients in both arms (approximately 74 %). The death rate in both arms after 14 months of follow-up was approximately 55 %. The TTF observed were greater in the FOLFIRI/ECX arm [29]. Table 1 presents the characteristics of patients.

**Table 1:** Characteristics of patients included in the study

Variables	ECX^a^/FOLFIRI arm	FOLFIRI/ECX arm
	*n = 209*	*n = 207*
Gender		
Men n (%)	154 (74)	155 (75)
Performance status at D_0_		
=0-1^b^ n (%)	175(83.7)	178(86.0)
=2 n (%)	34(16.3)	29(14.0)
Type of tumor		
Diffuse n (%)	46(22.0)	51(24.6)
Age (years)		
md^c^ ± inq	61 ± 16	61 ± 16
Follow-up (months)		
md ± inq	9 ± 12	9 ± 10
SAE^d^ n(%)		
After 7 evaluation time points	122 (58)	105 (51)
TTF^e^ (weeks)		
md ± inq	17 ± 20	22 ± 25
Deaths n(%)		
Global^f^	175 (84)	180 (87)
After 7 evaluation time points	116 (56)	113 (55)

^*a*^ *ECX: Epirubicin-Cisplatin-Capecitabine*

^*b*^ *Performance status*

*0 = Fully active, able to carry on all pre-disease performance / 1 = Restricted in physically strenuous activity but ambulatory and able to carry out work of a light or sedentary nature*

*2 = Ambulatory and capable of all selfcare but unable to carry out any work activities. Up and about more than 50 % of waking hours*

^*c*^ *median ± inter-quartile range*

^*d*^ *Serious adverse events*

^*e*^ *Time to therapeutic failure of the first-line treatment*

^*f*^ *Proportion of deaths whatever the therapeutic line*

We analyzed 1023 questionnaires from 364 patients, that is a mean of 3 (SD ± 2) (range 1–12) questionnaires per patient. Of the first 7 evaluations, 2 912 self-administered questionnaires (one score for the physical dimension) were expected given the total number of 416. Considering that for patients who died, the absence of a score after death was not MD, we counted 1 262 missing scores, which corresponds to a proportion of MD of approximately 43 %.

**Fig. 1:**
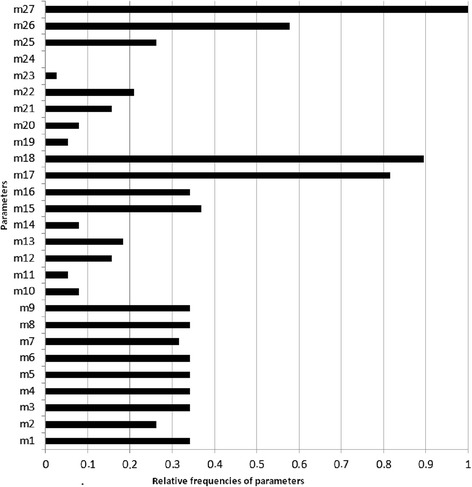
Distribution of the frequencies for the 27 parameters selected for optimal classification in 4 patterns. These parameters (m1 to m27) were statistical measures used to capture differences for a list of scores collected at different time points for each patient

With the process of maximizing the CritCF criterion, we retained the classification in 4 clusters and only 13 variability parameters (among the 27 initially proposed) listed in bold case in Additional file 1: Annex 3. These statistical parameters were selected as the most frequent as shown in Fig. 1. The corresponding CritCF mean value was 0.75 (range 0.61-0.87).

**Fig. 2:**
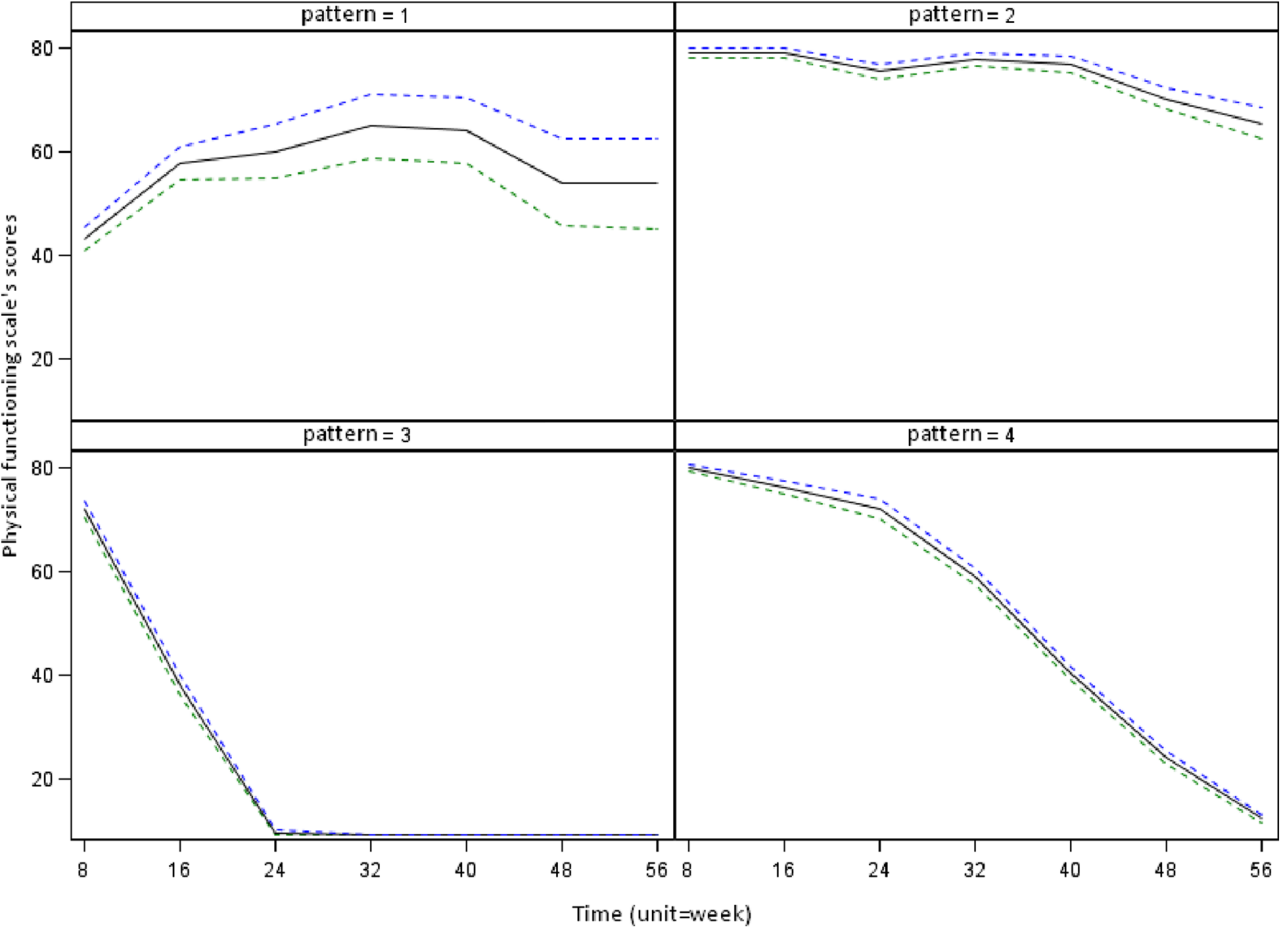
Representation of the quality of life change patterns over time

The 416 patients were definitively assigned to one of the 4 QoL change patterns P1, P2, P3 and P4 in the following proportions: 6 %, 41 %, 19 % and 34 %, respectively. Considering the MaxDiff value, we described a typology in 3 patterns: an improving pattern (P1) with MaxDiff = +25 pts, a stability pattern P2 (MaxDiff = −12pts) and 2 deterioration patterns P3 (MaxDiff = −21pts) and P4 (MaxDiff = −27pts). Figure 2 shows the trend for the mean scores per QoL change pattern over the 7 evaluation time points. These trend curves were bordered with curves showing the standard deviation of the mean scores. Each QoL change pattern was summarized using classical statistics computed from patients’ available data (age, gender, randomization arm, performance status, observed death rate and incidence of serious adverse events) as shown in Table 2.

**Table 2:** Characteristics of patients’ QoL change patterns

	Pattern 1*	Pattern 2	Pattern 3	Pattern 4	
					P^a^
Number of patients	24	171	78	143	
Gender					
Men n(%)	15 (63)	128 (75)	58 (74)	108 (76)	0.596
Age (years)					
md ± inq^b^	63 ± 17	61 ± 15	65 ± 19	60 ± 17	0.370
Performance status at D_0_					
=0-1^c^ n (%)	20 (83.3)	151 (88.3)	56 (71.8)	126 (88.1)	0.004
=2 n (%)	4 (16.7)	20 (11.7)	22 (28.2)	17 (11.9)	
Type of tumor					
Diffuse n (%)	7 (29.2)	33(19.3)	22(28.2)	35(24.5)	0.369
Randomization arm					
FOLFIRI n (%)	5 (20.8)	93 (54.4)	35 (44.9)	74 (51.8)	0.015
Quality of life Score					
Score_7_-Score_1_ (p^**^)	10.7 (0.520)	−13.4 (0.013)	−62.7 (0.038)	−67.6 (<10^−3^)	
SAE^d^					
n(%)	13 (54)	67 (39)	62 (81)	86 (60)	<10^−3^
TTF^e^ (weeks)					
md ± inq	15 ± 18	30 ± 24	4 ± 7	20 ± 12	<10^−3^
Death					
n(%)	3 (13)	18 (11)	78 (100)	131 (92)	<10^−3^

^*a*^ *: p for significance*

^*b*^ *:median ± inter-quartile range*

^*c*^ *Performance status*

*0 = Fully active, able to carry on all pre-disease performance / 1 = Restricted in physically strenuous activity but ambulatory and able to carry out work of a light or sedentary nature*

*2 = Ambulatory and capable of all selfcare but unable to carry out any work activities. Up and about more than 50 % of waking hours*

^*d*^ *Serious adverse events*

^*e*^ *time to therapeutic failure of the first-line treatment*

^*^ ***x-axis:*** *time (unit = weeks) and* ***y-axis:*** *the physical functioning scale scores (range [0–100])*

^**^ *p for significance of the slope predicted by a linear model*

**Fig. 3:**
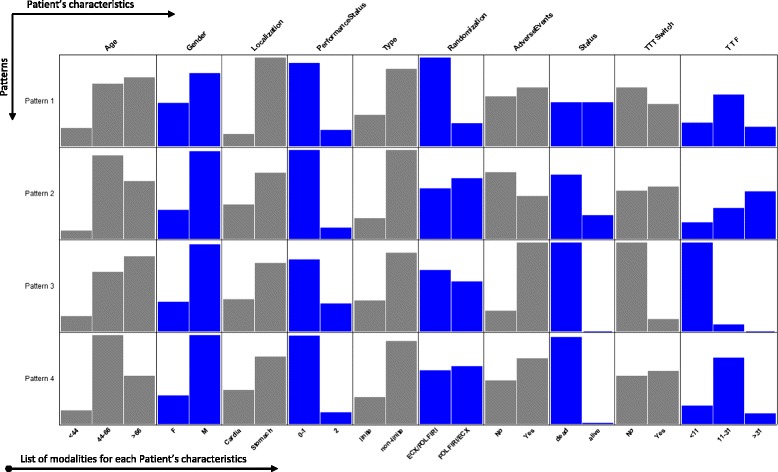
Description of the different change patterns using variables describing each patient individually

Figure 3 shows a much more visual description of the different patterns according to certain variables. The TTF variable associated with patients’ status and the information in Table 2 made it possible to characterize the 4 QoL change patterns. Pattern 1 mostly concerned patients from the ECX/FOLFIRI arm with equal proportions of living and dead patients. The TTF was between 11 and 31 weeks. In Pattern 2, we had relatively younger patients (age range [44–66] years old), mostly from FOLFIRI/ECX arm with the highest TTF (above 31 weeks). The majority of patient have died. For pattern 3, the majority of patients experienced serious adverse events and had the lowest TTF (less than 11 weeks), and pattern 4 essentially comprised patients with a TTF between 11 and 31 weeks. In Patterns 3 and 4, the majority of patients have died

**Fig. 4:**
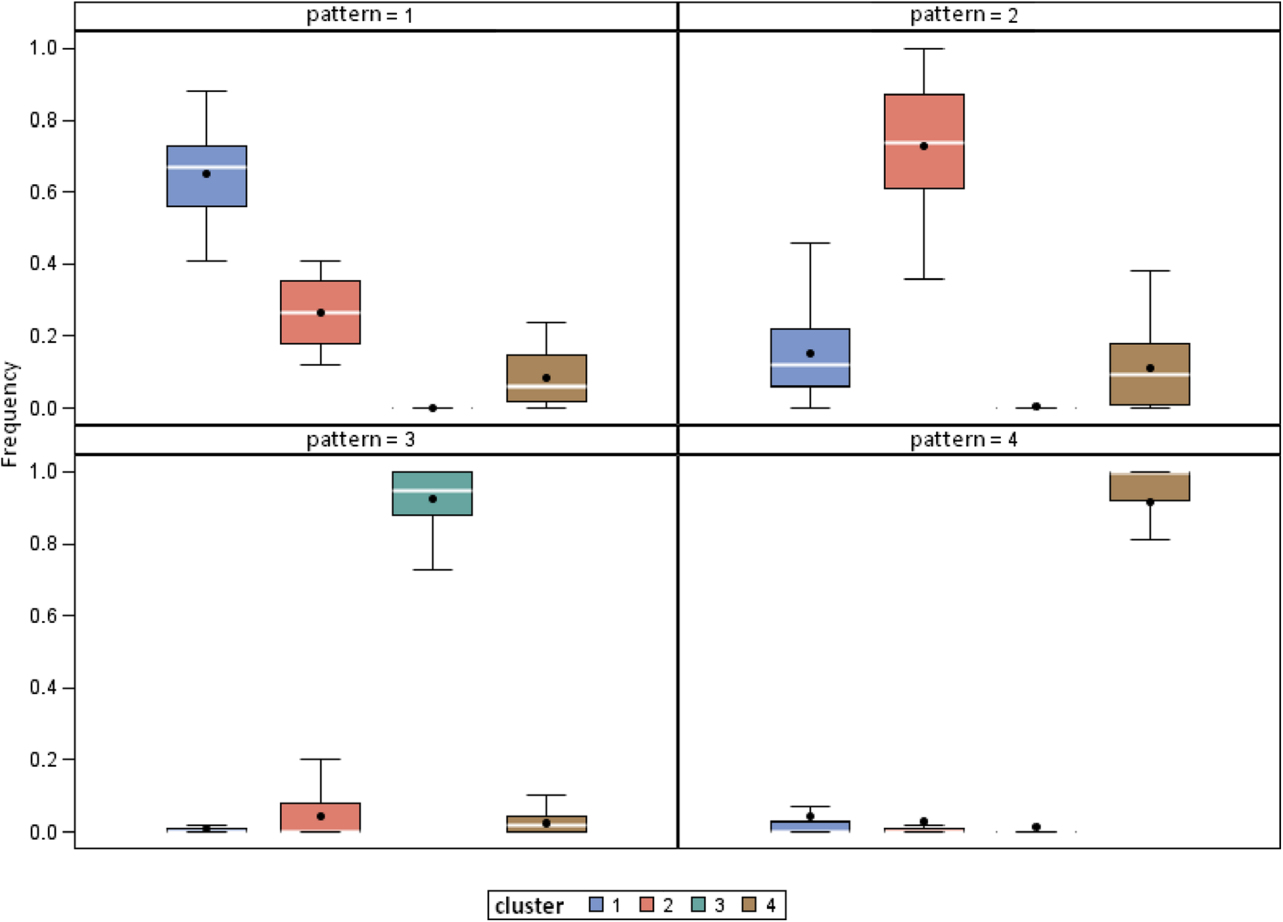
Visualization for each pattern with regard to the different clusters of patients’ individual score patterns (ISP)

The impact of the distribution of imputed values is represented in Fig. 4, which shows the distribution of the initial ISP clusters for each change pattern. Pattern 2, for example, comprised a majority of patients from cluster 2 (73 % on average), but also patients from cluster 1 for 15 % and more rarely patients from cluster 3. In the same way, the concordance coefficient between each of the 100 classifications and the final assignment was 0.62 (_95%_ CI [0.61-0.63]) on average.

## Discussion

In this work, 3 types of pattern were described: the improving pattern (P1), which had the smallest number of patients and a maximum difference (MaxDiff) between scores of +25 points; the stability pattern (P2), which included patients with a mean initial score equal to 80 and MaxDiff = −12 pts – this was the pattern with the most patients (41 %); and finally, two deterioration patterns (P3 and P4) can be distinguish according to the patients’ initial score: in the first group with a deterioration pattern (P3), with the smallest number of patients (78 versus 143), the initial QLQ-C30 physical score was around 43 while in the second (P4), it was around 82 on the 0–100 scale. And finally, we have proposed a graphical representation of these patterns. For the variables collected at inclusion, such as location, type of tumor, performance status and gender, we found that the structure of the bar chart was similar whatever the pattern. However, for the variable, age, the structure was nuanced with a predominance of the oldest patients in patterns P1 and P3, and relatively young patients were predominant in patterns P2 and P4. For variables collected throughout the study, such as serious adverse events (complications) or the time to therapeutic failure of the 1^st^ line therapy (TTF), the structure of the bar chart varied from one pattern to another.

Our methodological approach was different from the usual analysis of quality of life. In our work, we aim at defining distinct patterns of QoL evolution. Furthermore, we observe that each pattern was associated with a given clinical profile which confirms their pertinence, from a clinical point of view. Even if it was not the main aim of this paper, and that we did not use any prediction model, we think that these patterns of QoL may help clinicians to anticipate the course of QoL during cancer treatment according to the clinical profile. Our exploratory work would of course need to be confirmed by further results before any clinical use. Pooled clinical trials may provide a confirmation of the clinical features associated with the different patterns in a given diseases so that clinicians could use such profiles to identify patients with a low performance status at baseline but who could present a deterioration in their QoL. Patterns P3 and P4, for example, included patients with a low performance status (quite fully active all pre-disease performance) at baseline and the occurrence of serious adverse events (SAE) were associated with a physical QoL pattern of deterioration and a low baseline score. On the other hand, clinicians may wish to identify patients who could present an improvement in their QoL. For example, clinicians could select patients in the ECX arm with TTF mainly between 11 and 31 weeks as, in our study, we showed that patients classified in pattern P1 presented an improvement in their QoL compared with the other patterns. Clinicians may also want to know which patients will not have a major change in their QoL. In this case, they would be interested in selecting patients with TTF above 31 weeks (whatever the treatment arm) classified in pattern P2, which showed relative stability in our study. Of course, the results of this paper would need to be analyzed further before they could be used to try and predict the course of QoL during cancer treatment. Future analysis would assess the accuracy of the model that seeks to predict the course of QoL, based only on variables that are available at the time that the prediction is made. Statistics about predictive accuracy would be provided. Without such an analysis, it is difficult to ascertain the extent to which a predictive model would be useful.

Our results are in coherence with the literature. For example, Sadighi et al. [5] also found that patients who experienced a deterioration in their quality of life (pattern P3 in our study) also presented the shortest TTF (<11 weeks) and had been given ECX as the first-line treatment (ECX/FOLFIRI arm). In another paper, Curran et al. [4] also showed that patients who did not present a major change in their QoL (pattern P2 of stability in our study) had good clinical results (TTF above 31 weeks) and principally received irinotecan-based chemotherapy as the first-line treatment (FOLFIRI/ECX arm).

In this paper, we did not want to exclude patients who died or to consider them differently according to the cause of death.. In fact, in these types of clinical trials, the death rate is very high: most patients with locally advanced oeso-gastric cancer die before the first year, whatever the treatment. As a consequence, the analyses could not be performed after exclusion of these patients. For the main analysis, scores after death were set to zero. We are aware that this is a strong and maybe questionable hypothesis. Nonetheless, the same kind of assumption is stated when conducting time to degradation analyses (considering a major decrease of QoL as the same manner as death and pooling the two events).

It seems important to point out that these results should not eclipse the strategy used for missing data. Remember that for each missing score, the imputed values were chosen to represent both uncertainty about which values to impute assuming that both the reasons for non-response and uncertainty about the reasons for non-response are known. The principal advantage of using the multiple imputation method is the conservation of data distribution. In our study, this strategy led to a model that allowed a more realistic interpretation of a patient’s life, because it was less biased than the model we would have obtained using other imputation methods (using means, last available value, etc.). Nonetheless, this model still contains a certain number of pitfalls inherent to the construction of any statistical model. The advantage of this method was the fact that it produced patterns a priori independent from other clinical or personal characteristics. This means that it would be possible to analyze associations between these patterns and the study variables [16] or to search for predictive factors of these patterns. As a graphical representation makes it easier for clinicians to interpret the data, the presentation (bar charts) made it possible to visualize [41] the variability between the different patterns. This is sometimes difficult to grasp when it is reduced to standard deviations or confidence intervals alone.

## Conclusion

In this work we identified clinical profiles associated with QoL change over time from clinical trial results. Three main types of pattern were described: the improving pattern (P1), which had the smallest number of patients and a maximum difference (MaxDiff) between scores of +25 points; the stability pattern (P2), which included 41 % of patients, with a mean initial score equal to 80 and MaxDiff = −12 pts and finally, two deterioration patterns distinguished according to the patients’ initial score: in the first group (P3), the initial QLQ-C30 physical score was around 43 while in the second (P4), it was around 82 on the 0–100 scale. Our results have laid the foundations for deeper analyses (notably by studying the sensitivity of the results). In the future our methodology could be extended to the analyses of other scales of the EORTC-QLQ-C30 questionnaire, and to other therapeutic trials.

## Additional file

Additional file 1:
**Annex 1: The Full EORTC QLQ-C30 version 3 questionnaire with 30 items and their answer options.** The physical functioning scale is concerned by items 1 to 5. Annex 2: Construction steps of quality of life change pattern from longitudinal data score, taking into account the presence of missing scores. Annex 3: Listing of the 27 statistical measures of change with in bold case (N°), the measures selected for the classification. Annex 4: The CritCF formula. (DOCX 568 kb)
