# Supplementary material for: Construction of quality of life change patterns: example in oncology in a phase III therapeutic trial (FFCD 0307)
Source: Health Qual Life Outcomes. 2015 Sep 22;13:151. doi: 10.1186/s12955-015-0342-1 (PMC4578418; doi:10.1186/s12955-015-0342-1)
Supplement: Additional file 1: — Annex 1: The Full EORTC QLQ-C30 version 3 questionnaire with 30 items and their answer options. The physical functioning scale is concerned by items 1 to 5. Annex 2: Construction steps of quality of life change pattern from longitudinal data score, taking into account the presence of missing scores. Annex 3: Listing of the 27 statistical measures of change with in bold case (N°), the measures selected for the classification. Annex 4: The CritCF formula. (DOCX 568 kb) [file 12955_2015_342_MOESM1_ESM.docx]

**Annex 1: The Full EORTC QLQ-C30 version 3 questionnaire with 30 items and their answer options. The physical functioning scale is concerned by items 1 to 5**


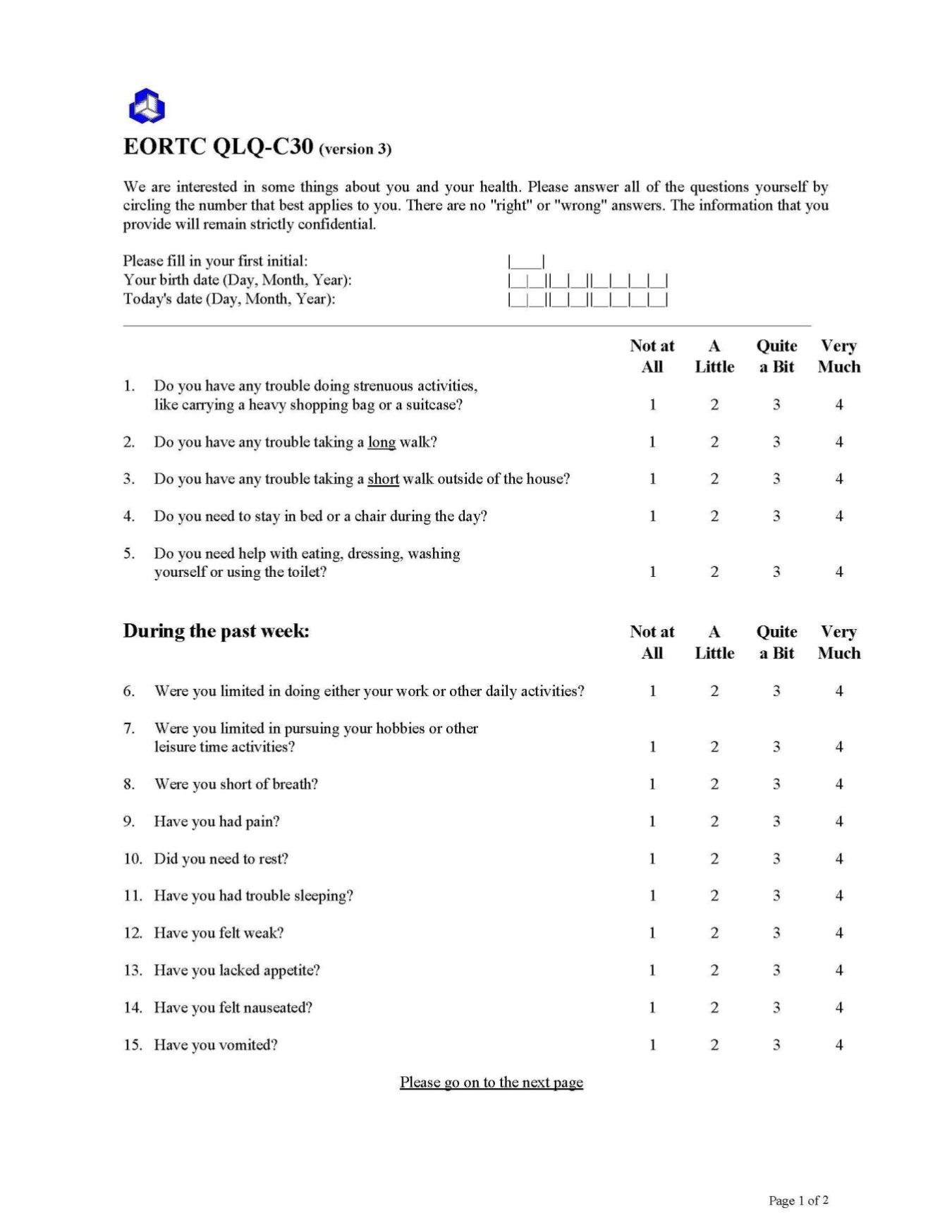


Items 1 to 15 of the EORTC QLQ-C30 questionnaire and their answer options.


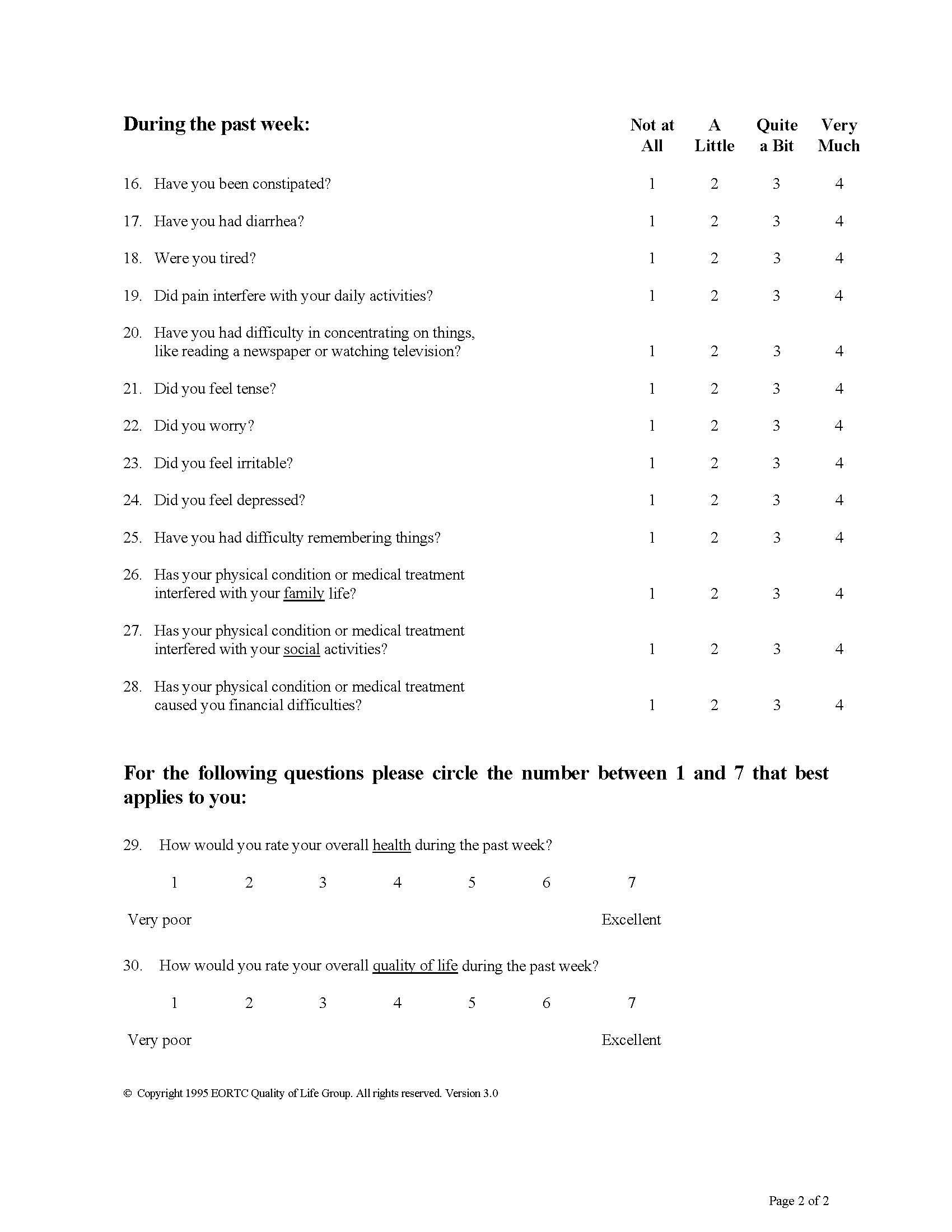


Items 16 to 30 of the EORTC QLQ-C30 questionnaire and their answer options

**Annex 2:**

| Oriiginal dataset | | | | | | | |
| --- | --- | --- | --- | --- | --- | --- | --- |
| id | T_1_ | T_2_ | T_3_ | T_4_ | T_5_ | T_6_ | T_7_ |
| 1 | 100 | 100 | 93,3 | . | 93,3 | . | . |
| 2 | 93,3 | 86,7 | 93,3 | 100 | 93,3 | 93,3 | 93,3 |
| 3 | 80 | . | 40 | 53,3 | . | 53,3 | . |
| 4 | . | 20,0 | . | . | . | . | . |
| 5 | 100 | . | 66,7 | 86,7 | . | 73,3 | 26,7 |
| … |  |  |  |  |  |  |  |
| Oriiginal dataset | | | | | | | |
| id | T_1_ | T_2_ | T_3_ | T_4_ | T_5_ | T_6_ | T_7_ |
| 1 | 100 | 100 | 93,3 | . | 93,3 | . | . |
| 2 | 93,3 | 86,7 | 93,3 | 100 | 93,3 | 93,3 | 93,3 |
| 3 | 80 | . | 40 | 53,3 | . | 53,3 | . |
| 4 | . | 20,0 | . | . | . | . | . |
| 5 | 100 | . | 66,7 | 86,7 | . | 73,3 | 26,7 |
| … |  |  |  |  |  |  |  |

| Imputation N°**100** | | | | | |
| --- | --- | --- | --- | --- | --- |
| id | T_1_ | T_2_ | … | T_6_ | T_7_ |
| 1 | 100 | 100 | … | **76,6** | **34.7** |
| 2 | 93,3 | 86,7 | … | 93,3 | 93.3 |
| 3 | 80 | **78,2**. | ... | 53,3 | **89.2** |
| 4 | **72,7** | 20,0 | ... | **6,70** | **93.3** |
| 5 | 100 | **71,7** | ... | 73,3 | 26.7 |
| … | … | …. | … | … | … |
| Imputation N°**100** | | | | | |
| id | T_1_ | T_2_ | … | T_6_ | T_7_ |
| 1 | 100 | 100 | … | **76,6** | **34.7** |
| 2 | 93,3 | 86,7 | … | 93,3 | 93.3 |
| 3 | 80 | **78,2**. | ... | 53,3 | **89.2** |
| 4 | **72,7** | 20,0 | ... | **6,70** | **93.3** |
| 5 | 100 | **71,7** | ... | 73,3 | 26.7 |
| … | … | …. | … | … | … |

| Imputation N°… | | | | | | | |
| --- | --- | --- | --- | --- | --- | --- | --- |
| id | T_1_ | T_2_ | … | | T_6_ | | T_7_ |
| 1 | 100 | 100 | … | | **76,6** | | **…** |
| 2 | 93,3 | 86,7 | … | | 93,3 | | … |
| 3 | 80 | **78,2**. | ... | | 53,3 | | **…** |
| 4 | **72,7** | 20,0 | ... | **6,70** | | **…** | |
| 5 | 100 | **71,7** | ... | | 73,3 | | … |
| … | … | …. | … | | … | | … |
| Imputation N°… | | | | | | | |
| id | T_1_ | T_2_ | … | | T_6_ | | T_7_ |
| 1 | 100 | 100 | … | | **76,6** | | **…** |
| 2 | 93,3 | 86,7 | … | | 93,3 | | … |
| 3 | 80 | **78,2**. | ... | | 53,3 | | **…** |
| 4 | **72,7** | 20,0 | ... | **6,70** | | **…** | |
| 5 | 100 | **71,7** | ... | | 73,3 | | … |
| … | … | …. | … | | … | | … |

| Imputation N°**2** | | | | | | |
| --- | --- | --- | --- | --- | --- | --- |
| id | T_1_ | T_2_ | … | T_6_ | | T_7_ |
| 1 | 100 | 100 | … | **81,8** | | **53.2** |
| 2 | 93,3 | 86,7 | … | 93,3 | | 93.3 |
| 3 | 80 | **78,2**. | ... | 53,3 | | **74.0** |
| 4 | **72,7** | 20,0 | ... | **60,7** | **26.7** | |
| 5 | 100 | **71,7** | ... | 73,3 | | 26.7 |
| … | … | …. | … | … | | … |
| Imputation N°**2** | | | | | | |
| id | T_1_ | T_2_ | … | T_6_ | | T_7_ |
| 1 | 100 | 100 | … | **81,8** | | **53.2** |
| 2 | 93,3 | 86,7 | … | 93,3 | | 93.3 |
| 3 | 80 | **78,2**. | ... | 53,3 | | **74.0** |
| 4 | **72,7** | 20,0 | ... | **60,7** | **26.7** | |
| 5 | 100 | **71,7** | ... | 73,3 | | 26.7 |
| … | … | …. | … | … | | … |

| Imputation N°**1** | | | | | |
| --- | --- | --- | --- | --- | --- |
| id | T_1_ | T_2_ | … | T_6_ | T_7_ |
| 1 | 100 | 100 | … | **90.5** | **95.6** |
| 2 | 93.3 | 86,7 | … | 93.3 | 93.3 |
| 3 | 80 | **78,2**. | ... | 53.3 | **93.3** |
| 4 | **72.7** | 20,0 | ... | **58.3** | **1.24** |
| 5 | 100 | **71,7** | ... | 73.3 | 26.7 |
| … | … | …. | … | … | … |
| Imputation N°**1** | | | | | |
| id | T_1_ | T_2_ | … | T_6_ | T_7_ |
| 1 | 100 | 100 | … | **90.5** | **95.6** |
| 2 | 93.3 | 86,7 | … | 93.3 | 93.3 |
| 3 | 80 | **78,2**. | ... | 53.3 | **93.3** |
| 4 | **72.7** | 20,0 | ... | **58.3** | **1.24** |
| 5 | 100 | **71,7** | ... | 73.3 | 26.7 |
| … | … | …. | … | … | … |

| Imputation N°**100** | | | | |
| --- | --- | --- | --- | --- |
| id | C_1_ | C_2_ | C_3_ | C_4_ |
| 1 | 0 | 1 | 0 | 0 |
| 2 | 1 | 0 | 0 | 0 |
| 3 | 0 | 0 | 1 | 0 |
| 4 | 0 | 0 | 0 | 1 |
| 5 | 0 | 0 | 1 | 0 |
| … | … | …. | … | … |
| Imputation N°**100** | | | | |
| id | C_1_ | C_2_ | C_3_ | C_4_ |
| 1 | 0 | 1 | 0 | 0 |
| 2 | 1 | 0 | 0 | 0 |
| 3 | 0 | 0 | 1 | 0 |
| 4 | 0 | 0 | 0 | 1 |
| 5 | 0 | 0 | 1 | 0 |
| … | … | …. | … | … |

| Imputation N°**…** | | | | |
| --- | --- | --- | --- | --- |
| id | C_1_ | C_2_ | C_3_ | C_4_ |
| 1 | 0 | 1 | 0 | … |
| 2 | 1 | 0 | 0 | … |
| 3 | 0 | 0 | 1 | … |
| 4 | 0 | 0 | 0 | … |
| 5 | 0 | 0 | 1 | … |
| … | … | …. | … | … |
| Imputation N°**…** | | | | |
| id | C_1_ | C_2_ | C_3_ | C_4_ |
| 1 | 0 | 1 | 0 | … |
| 2 | 1 | 0 | 0 | … |
| 3 | 0 | 0 | 1 | … |
| 4 | 0 | 0 | 0 | … |
| 5 | 0 | 0 | 1 | … |
| … | … | …. | … | … |

| Imputation N°**2** | | | | |
| --- | --- | --- | --- | --- |
| id | C_1_ | C_2_ | C_3_ | C_4_ |
| 1 | 0 | 0 | 0 | 1 |
| 2 | 1 | 0 | 0 | 0 |
| 3 | 0 | 0 | 1 | 0 |
| 4 | 0 | 0 | 0 | 1 |
| 5 | 0 | 0 | 1 | 0 |
| … | … | …. | … | … |
| Imputation N°**2** | | | | |
| id | C_1_ | C_2_ | C_3_ | C_4_ |
| 1 | 0 | 0 | 0 | 1 |
| 2 | 1 | 0 | 0 | 0 |
| 3 | 0 | 0 | 1 | 0 |
| 4 | 0 | 0 | 0 | 1 |
| 5 | 0 | 0 | 1 | 0 |
| … | … | …. | … | … |

| Imputation N°**1** | | | | |
| --- | --- | --- | --- | --- |
| id | C_1_ | C_2_ | C_3_ | C_4_ |
| 1 | 0 | 1 | 0 | 0 |
| 2 | 1 | 0 | 0 | 0 |
| 3 | 0 | 0 | 1 | 0 |
| 4 | 0 | 0 | 0 | 1 |
| 5 | 0 | 0 | 1 | 0 |
| … | … | …. | … | … |
| Imputation N°**1** | | | | |
| id | C_1_ | C_2_ | C_3_ | C_4_ |
| 1 | 0 | 1 | 0 | 0 |
| 2 | 1 | 0 | 0 | 0 |
| 3 | 0 | 0 | 1 | 0 |
| 4 | 0 | 0 | 0 | 1 |
| 5 | 0 | 0 | 1 | 0 |
| … | … | …. | … | … |

| id | C_1_ | C_2_ | C_3_ | C_4_ | CP |
| --- | --- | --- | --- | --- | --- |
| 1 | .98 | 0 | .02 | 0 | **1** |
| 2 | 0 | .24 | .74 | .02 | **3** |
| 3 | .12 | .32 | .28 | .28 | **2** |
| 4 | .16 | .18 | .44 | .22 | **3** |
| 5 | .1 | .32 | .36 | .22 | **2** |
| … | … | …. | … | … | … |

| Variability parameter's table per ISP | | | | | |
| --- | --- | --- | --- | --- | --- |
| Profils | P_1_ | P_2_ | … | P_26_ | P_27_ |
| PIS_1_ | X_1-1_ | X_1-2_ | … | X_1-26_ | X_1-27_ |
| PIS_2_ | X_2-1_ | X_2-2_ | … | X_2-26_ | X_2-27_ |
| PIS_3_ | X_3-1_ | X_3-2_ | … | X_3-26_ | X_3-27_ |
| PIS_4_ | X_4-1_ | X_4-2_ | … | X_4-26_ | X_4-27_ |
| PIS_5_ | X_5-1_ | X_5-2_ | … | X_5-26_ | X_5-27_ |
|  |  |  |  |  |  |
| Variability parameter's table per ISP | | | | | |
| Profils | P_1_ | P_2_ | … | P_26_ | P_27_ |
| PIS_1_ | X_1-1_ | X_1-2_ | … | X_1-26_ | X_1-27_ |
| PIS_2_ | X_2-1_ | X_2-2_ | … | X_2-26_ | X_2-27_ |
| PIS_3_ | X_3-1_ | X_3-2_ | … | X_3-26_ | X_3-27_ |
| PIS_4_ | X_4-1_ | X_4-2_ | … | X_4-26_ | X_4-27_ |
| PIS_5_ | X_5-1_ | X_5-2_ | … | X_5-26_ | X_5-27_ |
|  |  |  |  |  |  |

| Variability parameter's table per ISP | | | | | |
| --- | --- | --- | --- | --- | --- |
| Profils | P_1_ | P_2_ | … | P_26_ | P_27_ |
| PIS_1_ | X_1-1_ | X_1-2_ | … | X_1-26_ | X_1-27_ |
| PIS_2_ | X_2-1_ | X_2-2_ | … | X_2-26_ | X_2-27_ |
| PIS_3_ | X_3-1_ | X_3-2_ | … | X_3-26_ | X_3-27_ |
| PIS_4_ | X_4-1_ | X_4-2_ | … | X_4-26_ | X_4-27_ |
| PIS_5_ | X_5-1_ | X_5-2_ | … | X_5-26_ | X_5-27_ |
|  |  |  |  |  |  |
| Variability parameter's table per ISP | | | | | |
| Profils | P_1_ | P_2_ | … | P_26_ | P_27_ |
| PIS_1_ | X_1-1_ | X_1-2_ | … | X_1-26_ | X_1-27_ |
| PIS_2_ | X_2-1_ | X_2-2_ | … | X_2-26_ | X_2-27_ |
| PIS_3_ | X_3-1_ | X_3-2_ | … | X_3-26_ | X_3-27_ |
| PIS_4_ | X_4-1_ | X_4-2_ | … | X_4-26_ | X_4-27_ |
| PIS_5_ | X_5-1_ | X_5-2_ | … | X_5-26_ | X_5-27_ |
|  |  |  |  |  |  |

| Variability parameter's table per ISP | | | | | |
| --- | --- | --- | --- | --- | --- |
| Profils | P_1_ | P_2_ | … | P_26_ | P_27_ |
| ISP_1_ | X_1-1_ | X_1-2_ | … | X_1-26_ | X_1-27_ |
| PIS_2_ | X_2-1_ | X_2-2_ | … | X_2-26_ | X_2-27_ |
| PIS_3_ | X_3-1_ | X_3-2_ | … | X_3-26_ | X_3-27_ |
| PIS_4_ | X_4-1_ | X_4-2_ | … | X_4-26_ | X_4-27_ |
| PIS_5_ | X_5-1_ | X_5-2_ | … | X_5-26_ | X_5-27_ |
|  |  |  |  |  |  |
| Variability parameter's table per ISP | | | | | |
| Profils | P_1_ | P_2_ | … | P_26_ | P_27_ |
| ISP_1_ | X_1-1_ | X_1-2_ | … | X_1-26_ | X_1-27_ |
| PIS_2_ | X_2-1_ | X_2-2_ | … | X_2-26_ | X_2-27_ |
| PIS_3_ | X_3-1_ | X_3-2_ | … | X_3-26_ | X_3-27_ |
| PIS_4_ | X_4-1_ | X_4-2_ | … | X_4-26_ | X_4-27_ |
| PIS_5_ | X_5-1_ | X_5-2_ | … | X_5-26_ | X_5-27_ |
|  |  |  |  |  |  |

| Variability parameter's table per ISP | | | | | |
| --- | --- | --- | --- | --- | --- |
| Profils | P_1_ | P_2_ | … | P_26_ | P_27_ |
| ISP_1_ | X_1-1_ | X_1-2_ | … | X_1-26_ | X_1-27_ |
| ISP_2_ | X_2-1_ | X_2-2_ | … | X_2-26_ | X_2-27_ |
| ISP_3_ | X_3-1_ | X_3-2_ | … | X_3-26_ | X_3-27_ |
| ISP_4_ | X_4-1_ | X_4-2_ | … | X_4-26_ | X_4-27_ |
| ISP_5_ | X_5-1_ | X_5-2_ | … | X_5-26_ | X_5-27_ |
|  |  |  |  |  |  |
| Variability parameter's table per ISP | | | | | |
| Profils | P_1_ | P_2_ | … | P_26_ | P_27_ |
| ISP_1_ | X_1-1_ | X_1-2_ | … | X_1-26_ | X_1-27_ |
| ISP_2_ | X_2-1_ | X_2-2_ | … | X_2-26_ | X_2-27_ |
| ISP_3_ | X_3-1_ | X_3-2_ | … | X_3-26_ | X_3-27_ |
| ISP_4_ | X_4-1_ | X_4-2_ | … | X_4-26_ | X_4-27_ |
| ISP_5_ | X_5-1_ | X_5-2_ | … | X_5-26_ | X_5-27_ |
|  |  |  |  |  |  |

**Step 1** : Multiple imputation

**Step 2**: variability parameters computation

**Step 3** : Clustering

**Step 4**: Assigning a patient to one CP

*Id : Patient’s unique identification number*

*ISP :* individual score pattern

*T_1_, T_2_, …, T_7_ : Evaluating time for Quality of life*

*P_1_, P_2_, …, P_26_, P_27_ : Variability parameters computed for each ISP*

*C_1_, C_2_, …, C_4_ : Cluster of assignment*

*CP : Change pattern for each patientId : Patient’s unique identification number*

*ISP :* individual score pattern

*T_1_, T_2_, …, T_7_ : Evaluating time for Quality of life*

*P_1_, P_2_, …, P_26_, P_27_ : Variability parameters computed for each ISP*

*C_1_, C_2_, …, C_4_ : Cluster of assignment*

*CP : Change pattern for each patient*

**ISP for each patient patient 4ISP for each patient patient 4**

Construction steps of quality of life change pattern from longitudinal data score, taking into account the presence of missing scores

**Annex 3: Listing of the 27 statistical measures of change with in bold case (N°), the measures selected for the classification**

| **N°** | Measures of changes in y_i_ (i=1,…, k)^1^ | **Formula** |
| --- | --- | --- |
| **Elementary measures of change** | | |
| **1** | **range** | **max y_i_ – min y_i_** |
| 2 | Mean-over-time | $\bar{y}$=$\frac{1}{k}\sum_{i=1}^{k} y_{i}$ |
| **3** | **Standard deviation (SD)** | $\boldsymbol{S}_{\boldsymbol{y}}$**=**$\sqrt{\frac{\boldsymbol{1}}{\boldsymbol{k-1}}\left( \boldsymbol{y}_{\boldsymbol{i}}\boldsymbol{-}\bar{\boldsymbol{y}} \right)^{\boldsymbol{2}}}$ |
| **4** | **Coefficient of variation (CV)** | $\left( {\boldsymbol{S}_{\boldsymbol{y}}}/{\bar{\boldsymbol{y}}} \right)\boldsymbol{\times100}$ |
| **5** | **Change** | $\boldsymbol{y}_{\boldsymbol{k}}\boldsymbol{-}\boldsymbol{y}_{\boldsymbol{1}}$ |
| **6** | **Mean change per time unit** | $\left( \boldsymbol{y}_{\boldsymbol{k}}\boldsymbol{-}\boldsymbol{y}_{\boldsymbol{1}} \right)/\left( \boldsymbol{t}_{\boldsymbol{k}}\boldsymbol{-}\boldsymbol{t}_{\boldsymbol{1}}\boldsymbol{+1} \right)$ |
| 7 | Change relative to the first score | $\left( y_{k}-y_{1} \right)/{y_{1}}$ |
| **8** | **Change relative to the mean-over-time** | $\left( \boldsymbol{y}_{\boldsymbol{k}}\boldsymbol{-}\boldsymbol{y}_{\boldsymbol{1}} \right)/{\bar{\boldsymbol{y}}}$ |
| **9** | **Slope of the linear model** $\boldsymbol{y}_{\boldsymbol{i}}\boldsymbol{=}\boldsymbol{a}\boldsymbol{+}\boldsymbol{b}\boldsymbol{t}_{\boldsymbol{i}}\boldsymbol{+}\boldsymbol{\varepsilon}_{\boldsymbol{i}}$ | $\boldsymbol{b=}\frac{\sum_{\boldsymbol{i=1}}^{\boldsymbol{k}} \left( \boldsymbol{y}_{\boldsymbol{i}}\boldsymbol{-}\bar{\boldsymbol{y}} \right)\left( \boldsymbol{t}_{\boldsymbol{i}}\boldsymbol{-}\bar{\boldsymbol{t}} \right)}{\sum_{\boldsymbol{i=1}}^{\boldsymbol{k}} \left( \boldsymbol{t}_{\boldsymbol{i}}\boldsymbol{-}\bar{\boldsymbol{t}} \right)^{\boldsymbol{2}}}$ |
| 10 | Proportion of variance explained by the  linear model $y_{i}=a+bt_{i}+\varepsilon_{i}$ | $R^{2}=b^{2}\times\frac{\sum_{i=1}^{k} \left( t_{i}-\bar{t} \right)^{2}}{\sum_{i=1}^{k} \left( y_{i}-\bar{y} \right)^{2}}$ |
| **Measures of nonlinearity and of inconsistency of change, based on**  **the first differences (∆_(1,i)_=y_(i+1)_-y_i_)** | | |
| 11 | Maximum of the first differences | Max $\boldsymbol{\Delta}_{\boldsymbol{1,i}}$ |
| 12 | SD of the first differences | $S_{\Delta_{1}}=\sqrt{\frac{1}{k-2}\sum_{i=1}^{k-1} \left( \Delta_{1,i}-\bar{\Delta_{1}} \right)^{2}}$  Where $\bar{\Delta_{1}}$=$\frac{1}{k-1}\sum_{i=1}^{k-1} \Delta_{1,i}$ |
| 13 | SD of the first differences per time unit | $S_{{\Delta^{'}}_{1}}=\sqrt{\frac{1}{k-2}\sum_{i=1}^{k-1} \left( {\Delta^{'}}_{1,i}-{\bar{\Delta^{'}}}_{1} \right)^{2}}$  where ${\Delta^{'}}_{1,i}=\frac{\Delta_{1,i}}{t_{i+1}-t_{i}}$ |
| 14 | Mean of the absolute first differences | $\left\vert\bar{\Delta}_{1} \right\vert=\frac{1}{k-1}\sum_{i=1}^{k-1} \left\vert\Delta_{1,i} \right\vert$ |
| **15** | **Maximum of the absolute first differences** | **max**$\left\vert\boldsymbol{\Delta}_{\boldsymbol{1,i}} \right\vert$ |
| **16** | **Ratio of the maximum absolute first difference to the mean-over‑time** | $\left( \mathbf{max} \left\vert\boldsymbol{\Delta}_{\boldsymbol{1,i}} \right\vert\right)/{\bar{\boldsymbol{y}}}$ |
| **17** | **Ratio of the maximum absolute first difference to the slope** | $\left( \mathbf{max} \left\vert\boldsymbol{\Delta}_{\boldsymbol{1,i}} \right\vert\right)/\boldsymbol{b}$ |
| **18** | **Ratio of the SD of the first differences to the slope** | $\left( {\boldsymbol{S}_{\boldsymbol{\Delta}_{\boldsymbol{i}}}}/\boldsymbol{b} \right)$ |

^1^ *k* may vary from patient to patient

*(Continued …)*

| **N°** | Measures of changes in y_i_ (i=1,…, k)^1^ | **Formula** |
| --- | --- | --- |
| **Measures sensitive to nonmonotonicity and to abrupt short-term fluctuations, based on the second differences**  $\boldsymbol{\Delta}_{\boldsymbol{2}\boldsymbol{,}\boldsymbol{i}}\boldsymbol{=}\boldsymbol{\Delta}_{\boldsymbol{1}\boldsymbol{,}\boldsymbol{i}\boldsymbol{+}\boldsymbol{1}}\boldsymbol{-}\boldsymbol{\Delta}_{\boldsymbol{1}\boldsymbol{,}\boldsymbol{i}}\boldsymbol{=}\left( \boldsymbol{y}_{\boldsymbol{i}\boldsymbol{+}\boldsymbol{2}}\boldsymbol{-}\boldsymbol{y}_{\boldsymbol{i}\boldsymbol{+}\boldsymbol{1}} \right)\boldsymbol{-}\left( \boldsymbol{y}_{\boldsymbol{i}\boldsymbol{+}\boldsymbol{1}}\boldsymbol{-}\boldsymbol{y}_{\boldsymbol{i}} \right)\boldsymbol{=}\boldsymbol{y}_{\boldsymbol{i}\boldsymbol{+}\boldsymbol{2}}\boldsymbol{+}\boldsymbol{y}_{\boldsymbol{i}}\boldsymbol{-}\boldsymbol{2}\boldsymbol{y}_{\boldsymbol{i}\boldsymbol{+}\boldsymbol{1}}$ | | |
| 19 | Mean of the second differences | $\bar{\Delta_{2}}=\frac{1}{k-2}\sum_{i=1}^{k-2} \Delta_{2,i}$ |
| 20 | Mean of the absolute second differences | $\left\vert\bar{\Delta_{2}} \right\vert=\frac{1}{k-2}\sum_{i=1}^{k-2} \left\vert\Delta_{2,i} \right\vert$ |
| 21 | Maximum of the absolute second differences | Max $\left\vert\Delta_{2,i} \right\vert$ |
| 22 | Ratio of the maximum absolute second difference to the mean-over-time | $\left( \max\left\vert\Delta_{2,i} \right\vert\right)/\bar{y}$ |
| 23 | Ratio of the maximum absolute second difference to the mean absolute first difference | $\left( \max\left\vert\Delta_{2,i} \right\vert\right)/\left\vert\bar{\Delta_{1}} \right\vert$ |
| 24 | Ratio of the mean absolute second difference to the mean absolute first difference | $\left( \left\vert\bar{\Delta_{2}} \right\vert\right)/\left( \left\vert\bar{\Delta_{1}} \right\vert\right)$ |
| **Measures contrasting early vs. later change ^2^** | | |
| 25 | Ratio of early to later change | $\left( y_{c}-y_{1} \right)/\left( y_{k}-y_{c+1} \right)$ |
| **26** | **Ratio of early to total change** | $\left( \boldsymbol{y}_{\boldsymbol{c}}\boldsymbol{-}\boldsymbol{y}_{\boldsymbol{1}} \right)/\left( \boldsymbol{y}_{\boldsymbol{k}}\boldsymbol{-}\boldsymbol{y}_{\boldsymbol{1}} \right)$ |
| **27** | **Ratio of late to total change** | $\left( \boldsymbol{y}_{\boldsymbol{k}}\boldsymbol{-}\boldsymbol{y}_{\boldsymbol{c+1}} \right)/\left( \boldsymbol{y}_{\boldsymbol{k}}\boldsymbol{-}\boldsymbol{y}_{\boldsymbol{1}} \right)$ |

^1^ *k* may vary from patient to patient

^2^ Early and later changes are calculated for a given cut-point set between observations *c* and *c+*1.

**Annex 4: The CritCF formula**

The CritCF criterion is defined as

$CritCF=\left[ \left( \frac{2m}{\left( 2m+1 \right)} \right)*\left( \frac{1}{\left( 1+\frac{W}{B} \right)} \right) \right]^{\frac{{log}_{2}\left( k+1 \right)+1}{{log}_{2}\left( m+1 \right)+1}}$,

Where

*m= number of features*

*k=number of clusters*

*W= within cluster inertia*

*B=between cluster inertia*
